# Adjusting phosphate feeding regimen according to daily rhythm increases eggshell quality via enhancing medullary bone remodeling in laying hens

**DOI:** 10.1186/s40104-023-00829-0

**Published:** 2023-03-10

**Authors:** Jiakun Yan, Jiajie Wang, Jie Chen, Hao Shi, Xujie Liao, Chong Pan, Yanli Liu, Xin Yang, Zhouzheng Ren, Xiaojun Yang

**Affiliations:** College of Animal Science and Technology, Northwest A&F University, Yangling, 712100 Shaanxi China

**Keywords:** Body phosphorus rhythm, Bone remodeling, Eggshell formation, Laying hen, Phosphorus feeding regimen

## Abstract

**Background:**

Body phosphorus metabolism exhibits a circadian rhythm over the 24-h daily cycle. The egg laying behavior makes laying hens a very special model for investigating phosphorus circadian rhythms. There is lack of information about the impact of adjusting phosphate feeding regimen according to daily rhythm on the phosphorus homeostasis and bone remodeling of laying hens.

**Methods and results:**

Two experiments were conducted. In Exp. 1, Hy-Line Brown laying hens (*n* = 45) were sampled according the oviposition cycle (at 0, 6, 12, and 18 h post-oviposition, and at the next oviposition, respectively; *n* = 9 at each time point). Diurnal rhythms of body calcium/phosphorus ingestions and excretions, serum calcium/phosphorus levels, oviduct uterus calcium transporter expressions, and medullary bone (MB) remodeling were illustrated. In Exp. 2, two diets with different phosphorus levels (0.32% and 0.14% non-phytate phosphorus (NPP), respectively) were alternately presented to the laying hens. Briefly, four phosphorus feeding regimens in total (each included 6 replicates of 5 hens): (1) fed 0.32% NPP at both 09:00 and 17:00; (2) fed 0.32% NPP at 09:00 and 0.14% NPP at 17:00; (3) fed 0.14% NPP at 09:00 and 0.32% NPP at 17:00; (4) fed 0.14% NPP at both 09:00 and 17:00. As a result, the regimen fed 0.14% NPP at 09:00 and 0.32% NPP at 17:00, which was designed to strengthen intrinsic phosphate circadian rhythms according to the findings in Exp. 1, enhanced (*P* < 0.05) MB remodeling (indicated by histological images, serum markers and bone mineralization gene expressions), elevated (*P* < 0.05) oviduct uterus calcium transportation (indicated by transient receptor potential vanilloid 6 protein expression), and subsequently increased (*P* < 0.05) eggshell thickness, eggshell strength, egg specific gravity and eggshell index in laying hens.

**Conclusions:**

These results underscore the importance of manipulating the sequence of daily phosphorus ingestion, instead of simply controlling dietary phosphate concentrations, in modifying the bone remodeling process. Body phosphorus rhythms will need to be maintained during the daily eggshell calcification cycle.

**Graphical Abstract:**

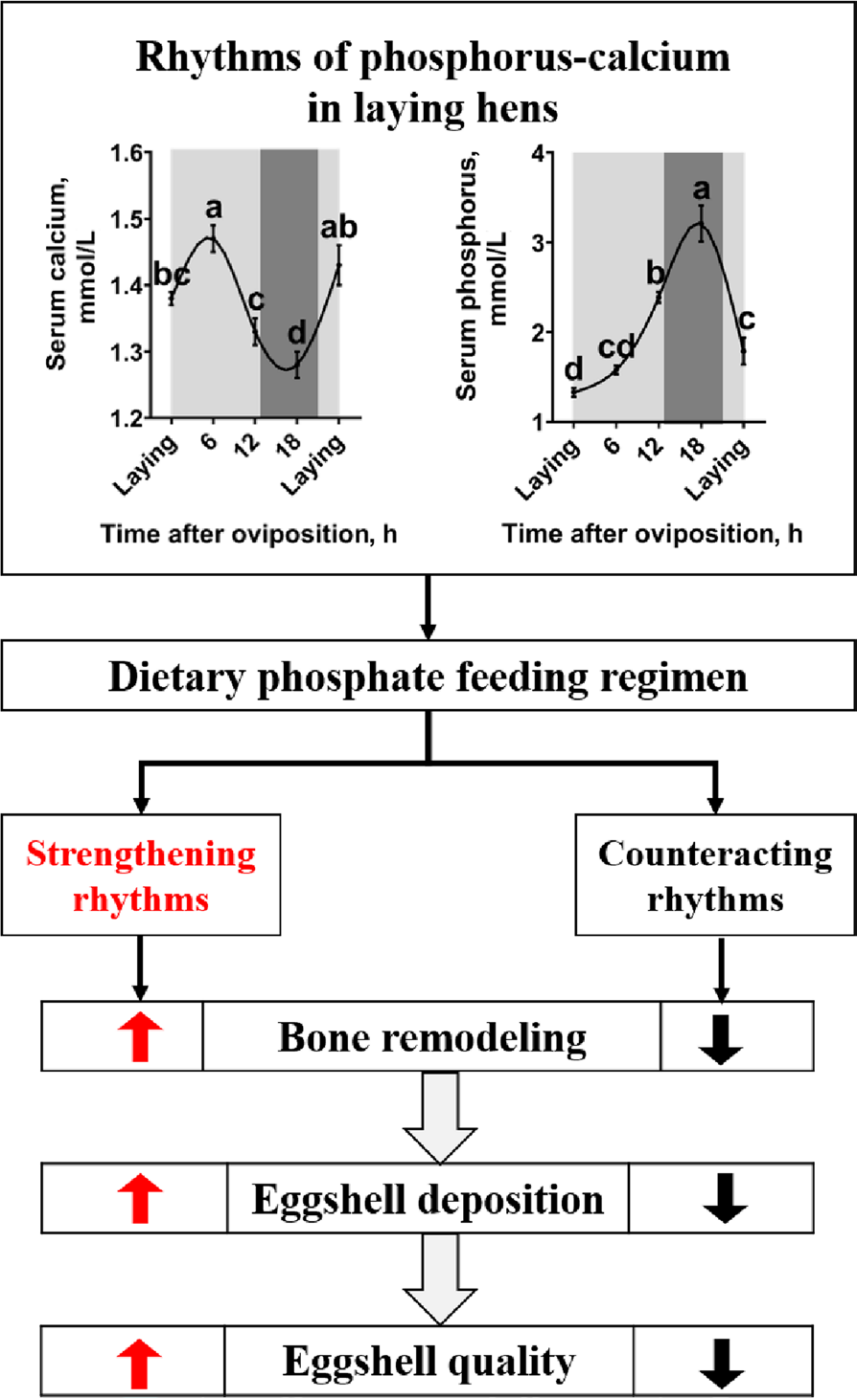

**Supplementary Information:**

The online version contains supplementary material available at 10.1186/s40104-023-00829-0.

## Introduction

Phosphate metabolism is involved in a variety of biologic processes [1] and its influx-storage-efflux balance is essential for life [2]. The two extremes of phosphate homeostasis (deficiency and excess) have been inextricably linked to tissue mineralization disorders including but not limit to osteopenia [3], osteoporosis [4], nephrocalcinosis [5] and vascular calcification [6]. Generally, dietary phosphate regimen must be precisely managed to maintain the balance of body phosphate homeostasis when problems occur during the intestinal absorption (phosphate influx) and/or bone mineralization (phosphate storage) and/or renal resorption (phosphate influx) processes [7]. In current practice, dietary phosphate regimen interventions are mostly conducted in two approaches: (1) direct controlling the concentrations of phosphate in ingested food [8]; (2) using phosphate binders (e.g., sevelamer carbonate) to remove available phosphate [9, 10] or using phosphate utilization promoters (e.g., calcitriol) to increase available phosphate [10, 11]. However, the effectiveness of these approaches has been questioned since the body exhibits significant diurnal variations in serum phosphate concentrations [12, 13], and a simple dietary intervention (which ignores the diurnal variations) may not enough for supporting a normal phosphate homeostasis [12, 13].

The phosphate metabolism exhibits a circadian rhythm over the 24-h daily cycle [14, 15]. Circadian rhythm disturbance of intestinal, renal and skeletal functions could induce abnormal phosphate rhythm and subsequently cause tissue mineralization disorders [16, 17]. For example, chronic kidney disease-mineral bone disorder, which exhibits a decreased serum phosphate circadian rhythm (amplitude decreases and phase shift) [18, 19], is often accompanied by circadian rhythm disturbances in the bone [20] and kidney [16]. In this case, simply increasing/decreasing dietary phosphate concentrations, could only overall increase/decrease serum phosphate concentrations, but could not normalize the circadian rhythm of serum phosphate [18]. These results indicating that the daily rhythms of body phosphate metabolism will need to be carefully maintained when developing dietary phosphate intervention strategies in humans and animals.

The egg laying behavior makes laying hens a very special model for investigating phosphate circadian rhythms [21]. Especially, the diurnal eggshell formation process in laying hens was support by a strong circadian rhythm in the remodeling of medullary bone (MB), which is unique to avian and dinosaurs and could be quickly absorbed and reformed during the 24-h egg-laying cycle [22]. It is well documented that circulating level of phosphate is highly dynamic during the MB cycle [21], and alterations in dietary phosphate levels could influence the remodeling of MB [23]. In aged laying hens, the remodeling efficiency of MB decreases and the frequency of cracked eggs increases (unqualified eggshell formation) [24]. In the field, attempts have been made to evaluate the most appropriate dietary phosphate levels for maintaining MB remodeling and increasing eggshell quality in aged laying hens [25]. However, in conventional feeding systems, laying hens are provided with a single diet with a constant phosphate level throughout the entire day without considering body phosphate circadian rhythms.

So, we hypothesized that a daily dynamic phosphate feeding regimen that enhancing body phosphate circadian rhythms would optimize the remodeling process of MB and thereby improve eggshell quality in laying hens. To test this hypothesis, we illustrated the circadian rhythms of phosphate metabolism and diets with different phosphate levels were alternately presented to the laying hens to meet the specific phosphate requirement during different stages of the daily egg-laying cycle. Our objectives were to reveal the multi-organ interactions on phosphate metabolism rhythms and to develop a simple daily phosphate regimen for improving eggshell quality in aged laying hens.

## Materials and methods

### Experimental animals, dosage regimen and sample collection

The animals used (Hy-Line Brown laying hens) were all purchased from Julong Poultry Farm (Wugong, Shaanxi, China), and were individually housed in cages with raised wire floors (depth × width × height = 45 cm × 35 cm × 45 cm) at the Animal Nutrition & Healthy Feeding Research Laboratory (Northwest A&F University, Yangling, China). A photoperiod of 16-h-light:8-h-dark was applied (lights-on, 05:30; lights-off, 21:30). The hens were fed twice daily (09:00 and 17:00).

### Exp. 1

Hy-Line Brown laying hens were fed with a regular diet (corn-soybean meal-based; containing 0.32% non-phytate phosphorus (NPP); Table 1) start from 35 weeks of age. On the last day of age 40 weeks, a total of 60 hens that laid eggs between 07:30−08:30 were randomly selected to evaluate the daily phosphorus rhythms. Of them, 45 hens were euthanized for sample collection, and the other 15 hens were used to study the feed intake and calcium/phosphorus excretion rhythms. For sample collection, the 45 hens were sampled according the oviposition cycle: at oviposition, at 6, 12, 18 h post-oviposition, and at the next oviposition, respectively, with 9 hens sampled at each of the time point. The following samples were collected: blood (for serum), uterine (stored at −80 ℃, for Western-blotting analysis), femur (in 4% paraformaldehyde, for histological analysis) and kidney (stored at −80 ℃, for Western-blotting analysis). For the other 15 hens, the feed intake was recoded and the excreta was collected at the following intervals: from oviposition to 6 h post-oviposition, from 7 to 12 h post-oviposition, from 13 to 18 h post-oviposition, from 19 h post-oviposition to the next oviposition.

**Table 1:** Composition and nutrient concentrations of basal diet (%, unless noted, as-is basis)

Item	Low phosphorus	Regular phosphorus
Ingredients		
Corn	56.69	56.69
Soybean meal	25.77	25.77
Distillers dried grains with solubles	4.00	4.00
Calcium carbonate	9.73	9.04
Dicalcium phosphate	-	1.15
Soybean oil	1.51	1.51
Sodium chloride	0.26	0.26
*DL*-Methionine	0.18	0.18
Choline chloride	0.15	0.15
Montmorillonite	0.71	0.25
Premix^1^	1	1
In total	100.00	100.00
Nutrient levels		
Metabolizable energy, kcal/kg (calculated)	2,600	2,600
Crude protein (calculated)	16.5	16.5
Total phosphorus (calculated/analyzed)	0.34/0.34	0.53/0.49
Non-phytate phosphorus (calculated)	0.14	0.32
Calcium (calculated/analyzed)	3.50/3.47	3.50/3.52

^1^Provided per kilogram of diet: manganese 60 mg, copper 8 mg, zinc 80 mg, iodine 0.35 mg, selenium 0.3 mg, vitamin A 8000 IU, vitamin E 30 mg, vitamin K_3_ 1.5 mg, thiamine 4 mg, riboflavin 13 mg, pantothenic acid 15 mg, nicotinamide 20 mg, pyridoxine 6 mg, biotin 0.15 mg, folic acid 1.5 mg, and cobalamin 0.02 mg

### Exp. 2

At the age of 70 weeks, a total of 120 hens were randomly selected to evaluate dietary interventions of body phosphorus rhythms. The hens were fed with 4 phosphorus regimens: (1) RR, provided with regular phosphorus diet at both 09:00 and 17:00 (conventional feeding without considering daily rhythms of body phosphorus metabolism); (2) RL, provided with regular phosphorus diet at 09:00 and low phosphorus diet at 17:00 (dynamic feeding converse to the body phosphorus rhythms found in Exp. 1); (3) LR, provided with low phosphorus diet at 09:00 and regular phosphorus diet at 17:00 (dynamic feeding consistent with the body phosphorus rhythms found in Exp. 1); (4) LL, provided with low phosphorus diet at both 09:00 and 17:00 (direct restriction without considering daily rhythms of body phosphorus metabolism). Each feeding regimens included 6 replicates, and each replicate contained 5 hens. The regular and the low phosphorus diet contained 0.32% and 0.14% NPP, respectively. The feeding trial lasted for 12 weeks (according to the literature, changes in eggshell and bone mineralization status could be observed in 8 to 12 weeks after dietary phosphorus interventions in laying hens) [26, 27]. On the last 3 d of the feeding trial, all the eggs were collected for egg quality analysis. On the last day of the feeding trial, two egg-laying hens were randomly selected from each replicate (sampled at 6 and 18 h post-oviposition, respectively). The following samples were collected: blood (for serum), uterine (stored at −80 ℃, for Western-blotting analysis ), femur (left side, stored in 4% paraformaldehyde for histological analysis; right side, stored at −80 ℃ for the determination of mineralization status and gene expressions).

### Serum biochemical assay

Blood samples (3 mL, from wing veins) were clotted at 37 ℃ for 60 min in water bath and centrifuged (594 g, 15 min) for serum samples (stored at −80 ℃). Serum levels of calcium (catalogue no. C004-2), phosphorus (catalogue no. C006-1) and tartrate-resistant acid phosphatase (TRAP; catalogue no. A058-1) were analyzed using commercial kits purchased from Nanjing Jiancheng Bioengineering Institute (Nanjing, Jiangsu, China). Serum levels of C-terminal telopeptide of type I collagen (CTX-I) was analyzed using a commercial kit (catalogue no. ml060903) purchased from Shanghai Enzyme-linked Biotechnology Co., Ltd (Shanghai, China). The spectrophotometric reactions were detected using a Synergy HT plate reader (BioTek, Winooski, VT, USA; for calcium and CTX-I analysis) or a UV-1800 spectrophotometer (Shimadzu, Kyoto, Japan; for phosphorus and TARP analysis).

### Determination of calcium and phosphorus contents

The excreta samples were oven dried, air equilibrated, and ground before analysis. The femur samples were cut at 25% and 75% from the proximal femur of the length of the bone to separate mid-diaphysis (50%), then this part was oven dried, defatted, cut longitudinally, and MB was removed by scraping with aid of scalpel. The pretreated samples were ashed using a muffle furnace (550 ℃; 6 h) and dissolved in hydrochloric acid. Calcium and phosphorus contents of the samples were determined using the ethylenediaminetetraacetic acid method and the ammonium-vanadium-molybdate method, respectively [28]. The results were calculated based on oven-dried-basis for excreta and ash-basis for femur samples.

### Histological analysis

The paraformaldehyde fixed femur samples were decalcified (6 weeks; in 10% EDTA phosphate buffer solution), dehydrated (in ethyl alcohol), hyalinized (in xylene), and the proximal diaphysis was embedded in paraffin wax. Sample sections (5 μm thick; mounted on glass slides) were stained with toluidine blue and scanned using an optical microscope (BX46; Olympus, Japan). Three photomicrographs (40× magnification) were taken from each sample. The area percentage of MB is expressed as the percentage of MB in the region of the marrow cavity where the MB is located (ImageJ 1.8.0. software).

### Western-blotting analysis

The protein isolation and western blotting procedures were conducted as previously described [29]. Primary antibodies (rabbit) to type 2a sodium-phosphate co-transporter (NPt2a, catalogue no. A9460), type III sodium-dependent phosphate transporter 1 (PiT1, catalogue no. A4117), type III sodium-dependent phosphate transporter 2 (PiT2, catalogue no. A6739), transient receptor potential vanilloid 6 (TRPV6, catalogue no. A16128), and calbindin D‐28k (CaBP-D28k, catalogue no. A0802) were purchased from ABclonal Technology (Wuhan, Hubei, China). Primary antibody (mouse) to β-actin (ACTB, catalogue no. CW0096) was purchased from CWBIO Co., Ltd. (Beijing, China). The secondary goat anti-rabbit IgG (catalogue no. DY60202) was purchased from Diyi Biotechnology Co., Ltd. (Shanghai, China) and the secondary goat anti-mouse IgG (catalogue no. bs-0296G-HRP) was purchased from Bioss Biotechnology Co., Ltd. (Beijing, China). The protein bands were visualized with a DNR imaging system (Micro Chemi, Israel) and the blot density was normalized to ACTB.

### Quantitative real-time PCR

The quantitative real‐time RCR analysis was performed as previously described [30]. The sequences (Table S1) of primers used in quantitative real-time PCR analysis were designed with the Primer3 program. All reactions were run in triplicate. Relative mRNA expressions were calculated using the chicken *ACTB* (β-actin) gene as an internal reference (2^−ΔΔCt^ method).

### Statistical analysis

Data analysis was performed using SPSS version 23.0 (IBM Corp., Chicago, IL, USA). The individual laying hen was considered as the statistical unit. Two-tailed Students’ *t*-tests was conducted for the comparisons between two groups. One-way ANOVA, followed by Duncan’s multiple-range post hoc test, was conducted to determine the differences among multiple groups. The results are presented as means and standard error of the mean (SEM). Statistical significance was considered at *P* < 0.05.

## Results

### Diurnal rhythms of serum calcium and phosphorus levels and uterine protein expressions in laying hens

**Fig. 1:**
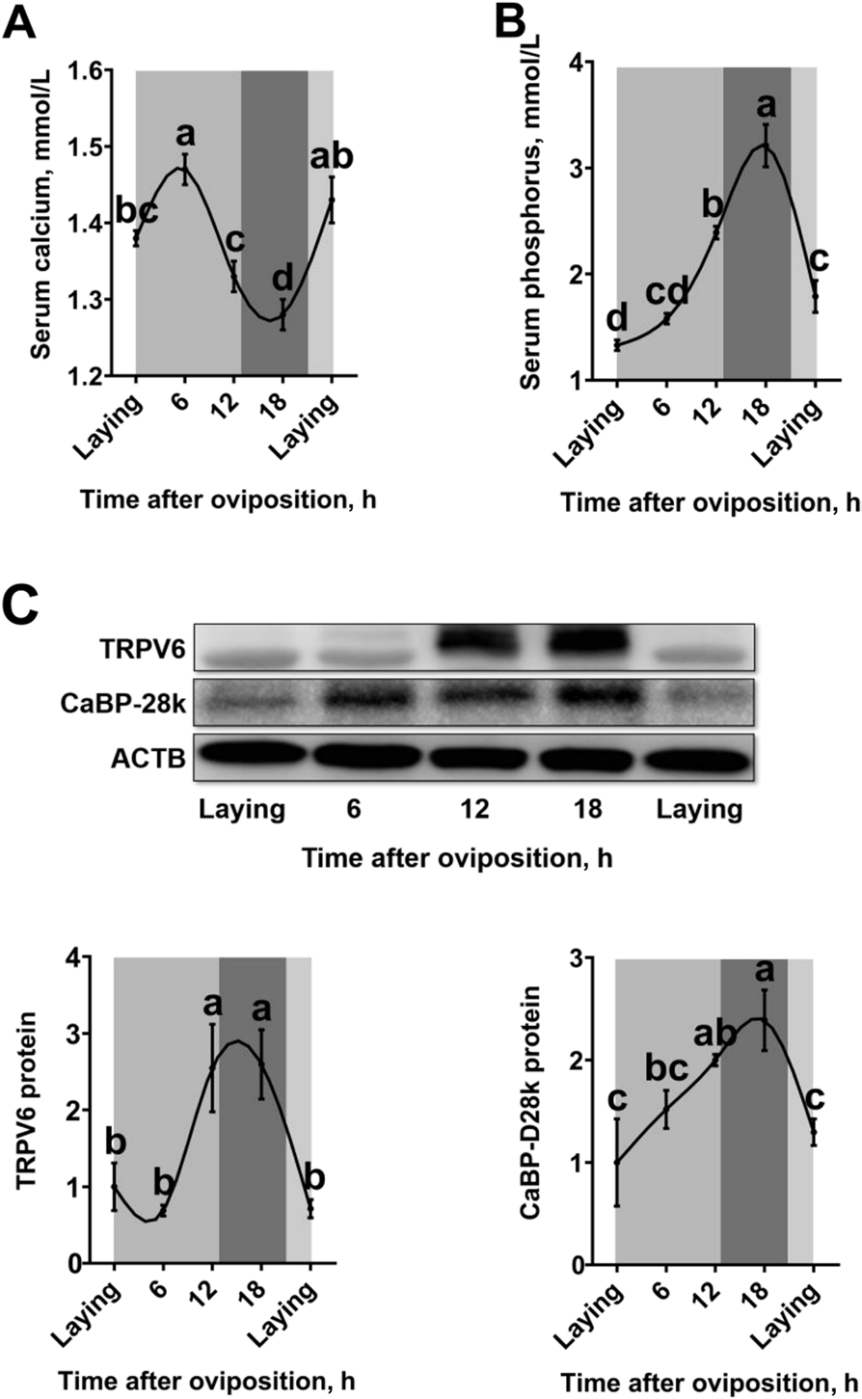
Diurnal rhythms of serum calcium and phosphorus levels and uterine protein expressions in 40-week-old Hy-Line Brown laying hens during the egg laying cycle. **A**) Serum calcium (*n* = 9 per group); **B**) serum phosphorus (*n* = 9 per group); **C**) protein expression of TRPV6 and CaBP-D28k (*n* = 4 per group). White and black bars represent the light and dark. Data are presented as the mean ± SEM. *P* < 0.05 by one-way ANOVA followed by Duncan's multiple range tests and the letters (a–d) indicate significant differences among all treatment groups (*P* < 0.05). ACTB, β-actin; CaBP-D28k, Calbindin D‐28k; TRPV6, Transient receptor potential vanilloid 6

Serum calcium level of the laying hens was increased after oviposition and peaked at 6 h post-oviposition (Fig. 1A, *P *< 0.05). Then, the serum calcium level was gradually decreased to its lowest level at 18 h post-oviposition (*P* < 0.05). Thereafter, increased at the next oviposition (*P* < 0.05). Serum phosphorus level (Fig. 1B) and uterine CaBP-D28k expression (Fig. 1C) of the laying hens were gradually increased after oviposition (*P* < 0.05), peaked at 18 h post-oviposition, and then deceased at the next oviposition (*P* < 0.05). Uterine expression of TRPV6 was higher at 12 h and 18 h post-oviposition (the period of eggshell fast deposition) when compared to the other time points during the egg laying cycle (Fig. 1C,  *P *< 0.05).

### Diurnal rhythms of MB remodeling in laying hens

**Fig. 2:**
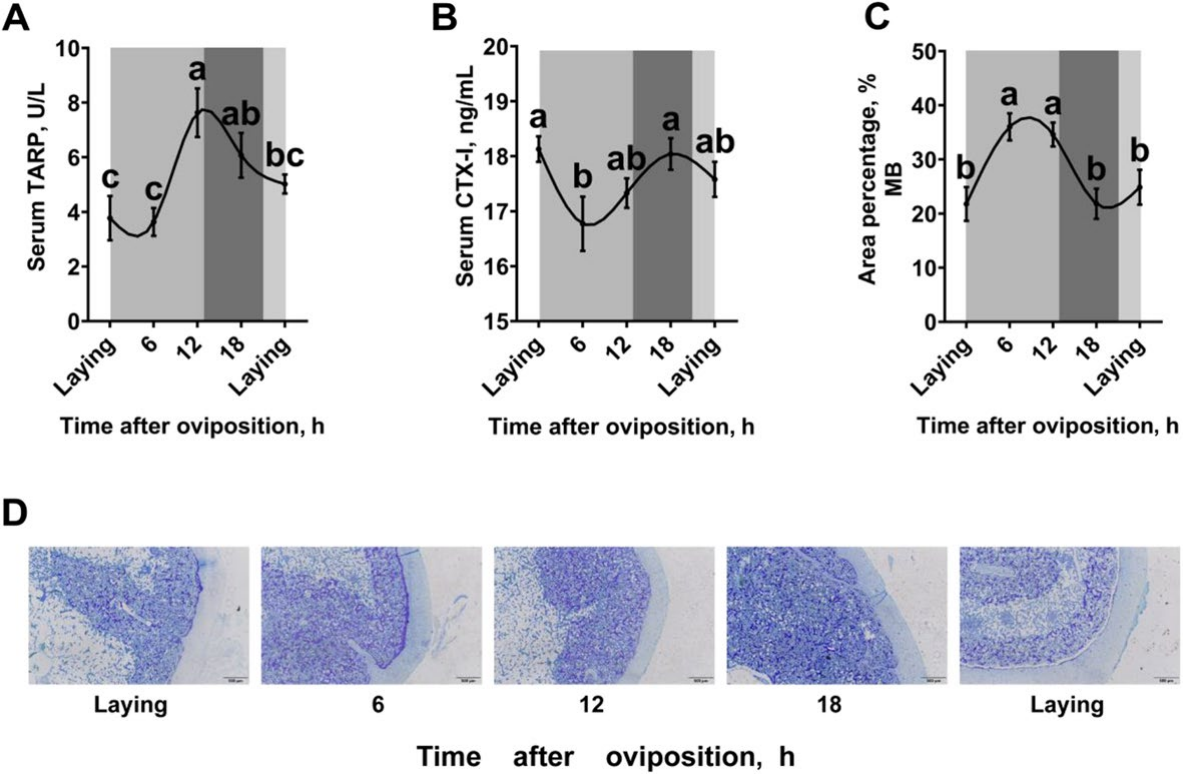
Diurnal rhythms of MB remodeling in 40-week-old Hy-Line Brown laying hens. **A**) Serum TARP (*n* = 9 per group); **B**) serum CTX-I (*n* = 9 per group); **C**) area percentage of MB analyzed by toluidine blue staining (*n* = 3 per group); **D**) representative images of toluidine blue staining of femur transverse sections, bar represents 100 µm. White and black bars represent the light and dark. Data are presented as the mean ± SEM. *P* < 0.05 by one-way ANOVA followed by Duncan's multiple range tests and the letters (a–c) indicate significant differences among all treatment groups (*P* < 0.05). CTX-I, C-terminal telopeptide of type I collagen; MB, medullary bone; TRAP, tartrate-resistant acid phosphatase

Serum concentrations of bone resorption markers TRAP (Fig. 2A) and CTX-I (Fig. 2B) were significantly changed (*P* < 0.05) during the daily egg laying cycle, and peaked at 12 h and 18 h post-oviposition (the period of eggshell fast deposition), respectively. The area percentage of MB was higher (*P* < 0.05) at 6 h and 12 h post-oviposition when compared to the other time points during the egg laying cycle (Fig. 2C, D).

### Diurnal rhythms of the intake and excretion of calcium and phosphorus in laying hens

**Fig. 3:**
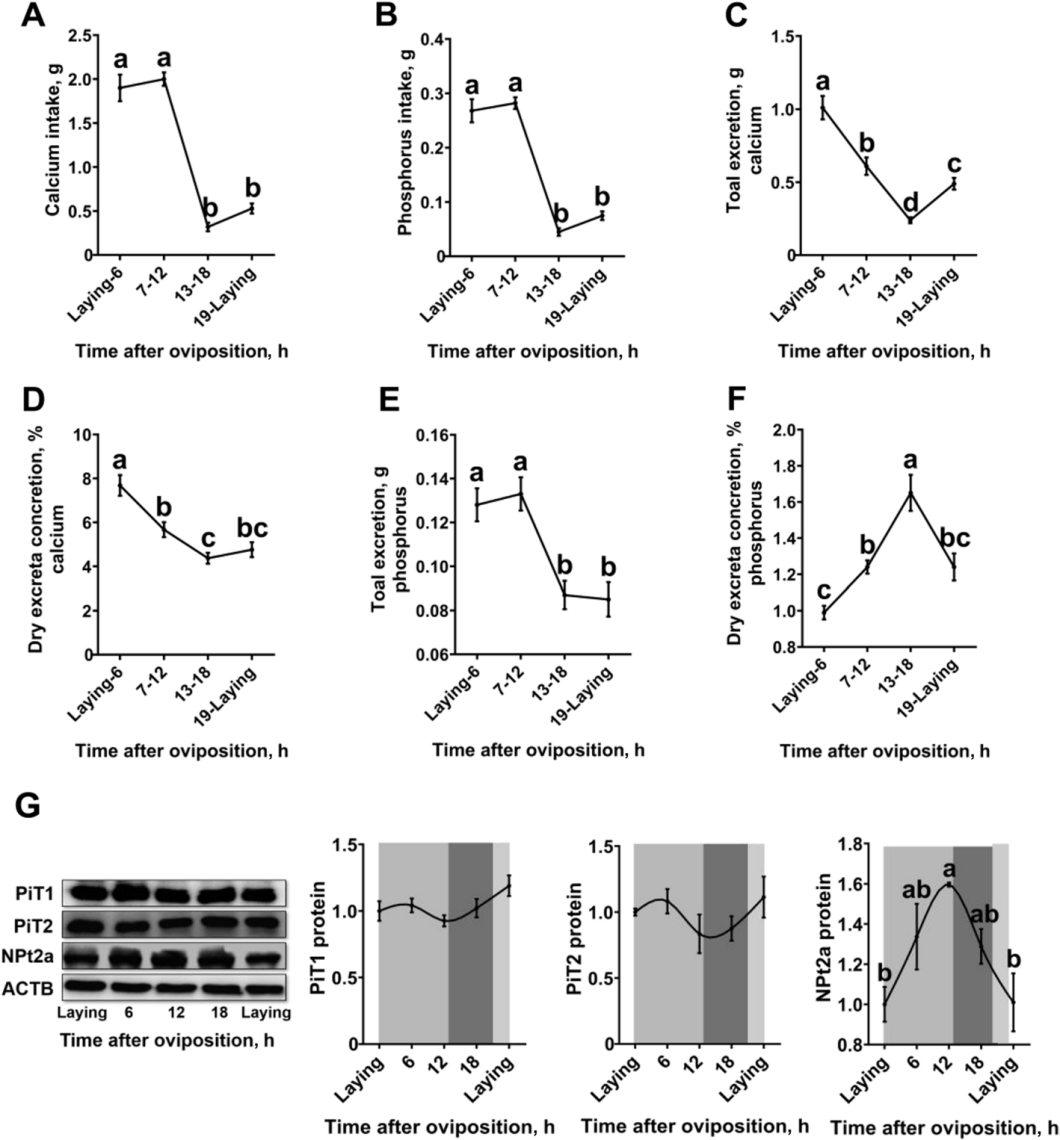
Diurnal rhythms of the intake and excretion of calcium and phosphorus in 40-week-old Hy-Line Brown laying hens. **A**) Calcium intake (*n* = 15 per group); **B**) phosphorus intake (*n* = 15 per group); **C**) total excretion of calcium (*n* = 15 per group); **D**) dry excrete concretion of calcium (*n* = 15 per group); **E**) total excretion of phosphorus (*n* = 15 per group); **F**) dry excrete concretion of phosphorus (*n* = 15 per group); **G**) representative western blots and statistical analysis of protein abundances of ACTB, PiT1, PiT2 and NPt2a in the kidney (*n* = 4 per group) , all samples were normalized to their respective ACTB levels of each sample. White and black bars represent the light and dark. Data are presented as the mean ± SEM. *P* < 0.05 by one-way ANOVA followed by Duncan's multiple range tests and the letters (a–d) indicate significant differences among all treatment groups (*P* < 0.05). ACTB, β-actin; NPt2a, Type 2a sodium-phosphate co-transporters; PiT1, Type III sodium-dependent phosphate transporter 1; PiT2, Type III sodium-dependent phosphate transporter 2

Calcium and phosphorus intake of the laying hens were majorly happened during the lighting period (Fig. 3A, B). The excreta collected during 0 to 6 h post-oviposition had increased (*P* < 0.05) calcium concentration when compared to those collected from the other period during the egg laying cycle (Fig. 3C). The excreta collected during 7 to 12 h post-oviposition had increased (*P* < 0.05) calcium concentration when compared to those collected during 13 to 18 h post-oviposition. Accordingly, body calcium excretion was the highest during 0 to 6 h post-oviposition and was the lowest during 13 to 18 h post-oviposition (Fig. 3D, *P *< 0.05). Body phosphorus excretion was majorly happened during the lighting period (Fig. 3E). The excreta collected during 13 to 18 h post-oviposition had the highest phosphorus concentration and the excreta collected during 0 to 6 h post-oviposition had the lowest phosphorus concentration (Fig. 3F, *P *< 0.05). Kidney expression of NPt2a of the laying hens was gradually increased after oviposition (Fig. 3G, *P* < 0.05), peaked at 12 h post-oviposition, and then deceased all the way down until the next oviposition (*P* < 0.05).

### Daily dynamic phosphorus feeding regimen increased uterine calcium transportation and eggshell quality in laying hens

**Fig. 4:**
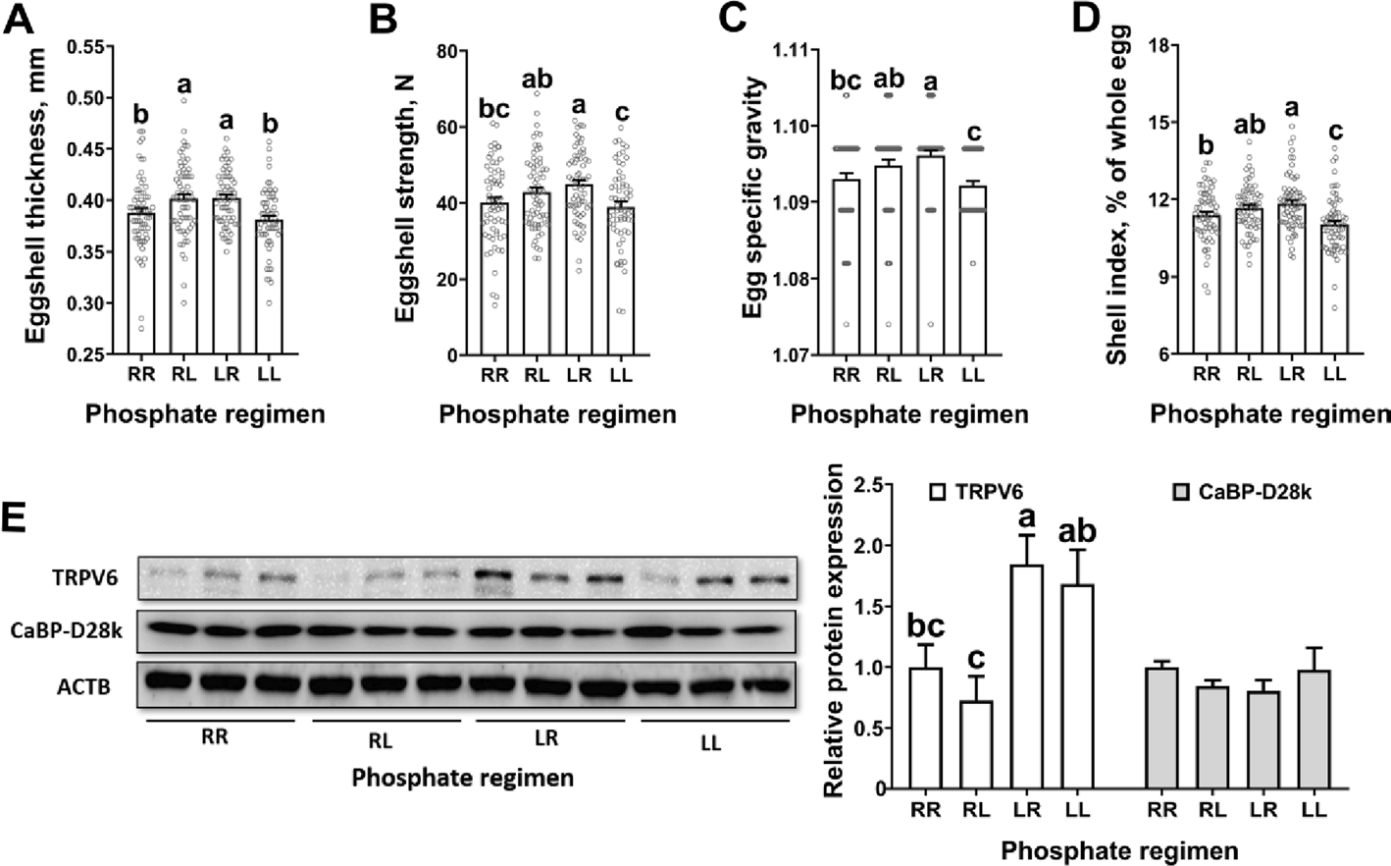
Daily dynamic phosphorus feeding regimen increased uterine calcium transportation and eggshell quality in 70-week-old Hy-Line Brown laying hens for 12 weeks. **A**) Eggshell thickness (*n* = 62−66 per group); **B**) eggshell strength (*n* = 62−66 per group); **C**) egg specific gravity (*n* = 61−66 per group); **D**) shell index (*n* = 62−63 per group); **D**) western blot analysis and statistical analysis of protein abundances of ACTB, TRPV6 and CaBP-D28k in the uterus collected from 18 h post-oviposition (*n* = 3 per group), all samples were normalized to their respective ACTB levels of each sample. Data are presented as the mean ± SEM. *P* < 0.05 by one-way ANOVA followed by Duncan's multiple range tests and the letters (a−c) indicate significant differences among all treatment groups (*P* < 0.05). RR, provided with regular phosphorus diet at both 09:00 and 17:00 for 12 weeks; RL, provided with regular phosphorus diet at 09:00 and low phosphorus diet at 17:00 for 12 weeks; LR, provided with low phosphorus diet at 09:00 and regular phosphorus diet at 17:00 for 12 weeks; LL, provided with low phosphorus diet at both 09:00 and 17:00 for 12 weeks. ACTB, β-actin; CaBP-D28k, Calbindin D‐28k; TRPV6, Transient receptor potential vanilloid 6

Laying hens on the RL and LR phosphorus feeding regimen had increased eggshell thickness (*P* < 0.05) when compared to those on the regimens of RR and LL (Fig. 4A). Laying hens on the LR phosphorus feeding regimen had: (1) increased (*P* < 0.05) eggshell strength, egg specific gravity and eggshell index when compared to those on the regimens of RR and LL (Fig. 4B−D); (2) increased (*P* < 0.05) uterine TRPV6 expression when compared to those on the regimens of RR and RL (Fig. 4E). Laying hens on the RL phosphorus feeding regimen had: (1) increased (*P* < 0.05) eggshell strength, egg specific gravity and eggshell index when compared to those on the LL regimen (Fig. 4B−D); (2) decreased (*P* < 0.05) uterine TRPV6 expression when compared to those on the regimens of LL (Fig. 4E).

### Daily dynamic phosphorus feeding regimen improved MB remodeling in laying hens

**Fig. 5:**
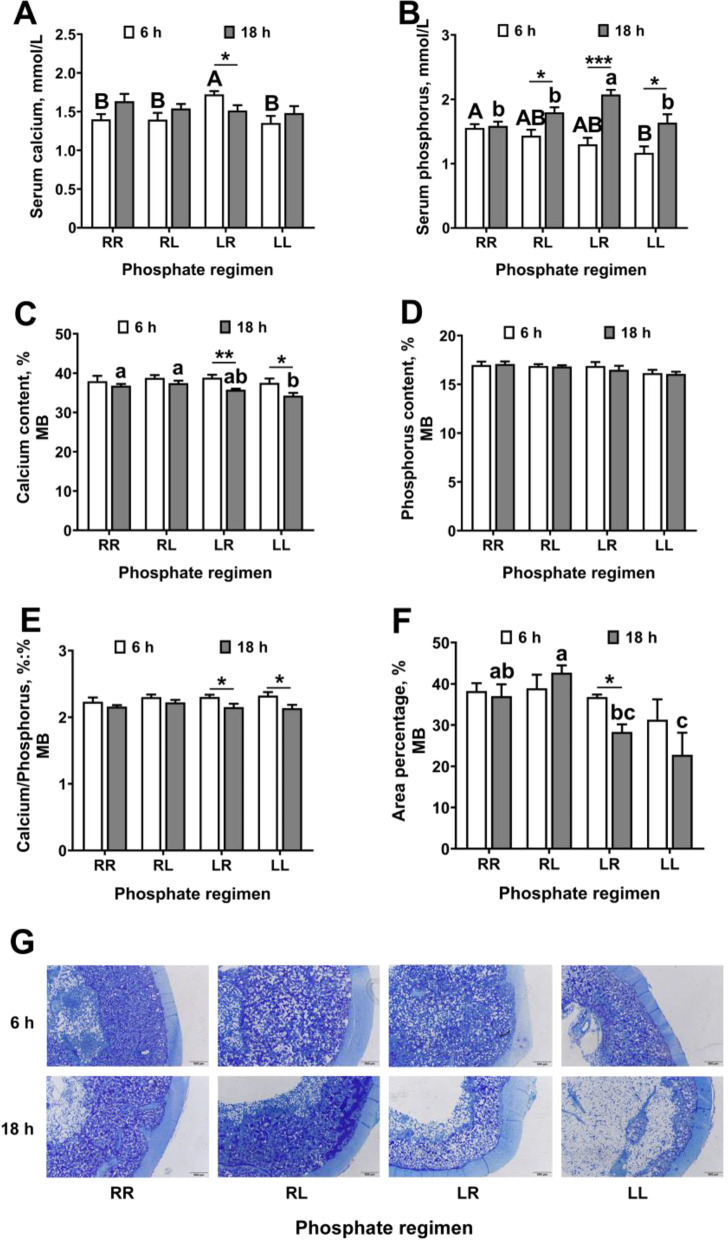
Daily dynamic phosphorus feeding regimen improved MB remodeling in 70-week-old Hy-Line Brown laying hens for 12 weeks. **A**) serum calcium (*n* = 6 per group); **B**) serum phosphorus (*n* = 6 per group); **C**) calcium content of MB (*n* = 6 per group); **D**) phosphorus content of MB (*n* = 6 per group); **E**) calcium/phosphorus ratio of MB (*n* = 6 per group); **F**) area percentage of MB analyzed by toluidine blue staining (*n* = 3 per group); **G**) representative images of toluidine blue staining of femur transverse sections, bar represents 100 µm. Data are presented as the mean ± SEM. *P* < 0.05 by one-way ANOVA followed by Duncan's multiple range tests, the letters (A, B) and the letters (a, b) indicate significant differences among all treatment groups chosen from 6 h post-oviposition and 18 h post-oviposition, respectively (*P* < 0.05). The significance of difference between 6 h and 18 h post-oviposition for each group was analyzed using two-tailed students’ *t*-tests, ^*^P < 0.05, ^**^P < 0.01, ^***^P < 0.001. RR, provided with regular phosphorus diet at both 09:00 and 17:00 for 12 weeks; RL, provided with regular phosphorus diet at 09:00 and low phosphorus diet at 17:00 for 12 weeks; LR, provided with low phosphorus diet at 09:00 and regular phosphorus diet at 17:00 for 12 weeks; LL, provided with low phosphorus diet at both 09:00 and 17:00 for 12 weeks; MB, medullary bone

At 6 h post-oviposition: (1) laying hens on the LR phosphorus feeding regimen had increased serum calcium (Fig. 5A, *P *< 0.05) when compared to those on the RR, RL and LL regimens; (2) laying hens on the RR phosphorus feeding regimen had increased serum phosphorus (Fig. 5B, *P *< 0.05) when compared to those on the LL regimen. At 18 h post-oviposition: (1) laying hens on the RR and RL phosphorus feeding regimens had increased MB calcium content (Fig. 5C, *P* < 0.05) when compared to those on LL regimen; (2) laying hens on the RL phosphorus feeding regimen had increased MB percentage (Fig. 5F, G, *P *< 0.05) when compared to those on the LR and LL regimens; (3) laying hens on the LR phosphorus feeding regimen had increased serum phosphorus (Fig. 5B, *P *< 0.05) when compared to those on the RR, RL and LL regimens. Within each feeding regimen: (1) laying hens on the RL phosphorus feeding regimen had increased serum phosphorus (Fig. 5B, *P *< 0.05) at 18 h post-oviposition when compared to 6 h post-oviposition; (2) laying hens on the LR phosphorus feeding regimen had decreased serum calcium (Fig. 5A, *P *< 0.05), MB calcium content (Fig. 5C, *P* < 0.05), MB calcium/phosphorus ratio (Fig. 5E, *P *< 0.05), and MB percentage (Fig. 5F, G, *P*< 0.05), and increased serum phosphorus (Fig. 5B, *P *< 0.05) at 18 h post-oviposition when compared to 6 h post-oviposition; (3) laying hens on the LL phosphorus feeding regimen had increased serum phosphorus (Fig. 5B, *P *< 0.05), and decreased MB calcium content (Fig. 5C, *P *< 0.05) and MB calcium/phosphorus ratio (Fig. 5E, *P *< 0.05) at 18 h post-oviposition when compared to 6 h post-oviposition. No difference was observed among treatments on MB phosphorus content (Fig. 5D, *P *> 0.05).

**Fig. 6:**
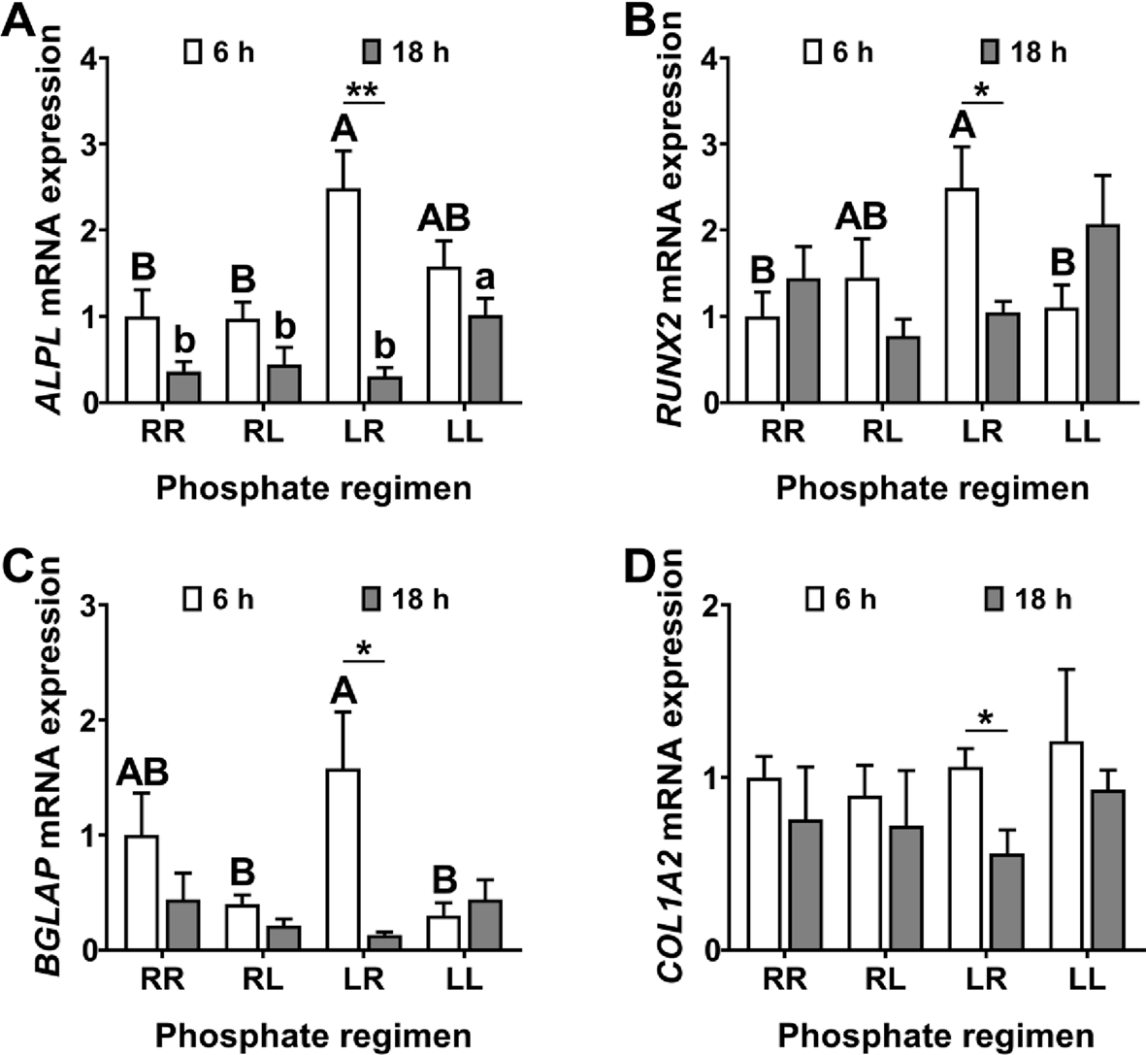
Daily dynamic phosphorus feeding regimen improved the rhythm of MB mineralization markers in 70-week-old Hy-Line Brown laying hens. **A**) *ALPL* mRNA (*n* = 6 per group); **B**) *RUNX2* mRNA (*n* = 6 per group); **C**) *BGLAP* mRNA (*n* = 6 per group); **D**) *SLC1A2* mRNA (*n* = 6 per group). Data are presented as the mean ± SEM. *P* < 0.05 by one-way ANOVA followed by Duncan's multiple range tests, the letters (A, B) and the letters (a, b) indicate significant differences among all treatment groups chosen from 6 h post-oviposition and 18 h post-oviposition, respectively (*P* < 0.05). The significance of difference between 6 h and 18 h post-oviposition for each group was analyzed using two-tailed students’ *t*-tests, ^*^P < 0.05, ^**^P < 0.01. RR, provided with regular phosphorus diet at both 09:00 and 17:00 for 12 weeks; RL, provided with regular phosphorus diet at 09:00 and low phosphorus diet at 17:00 for 12 weeks; LR, provided with low phosphorus diet at 09:00 and regular phosphorus diet at 17:00 for 12 weeks; LL, provided with low phosphorus diet at both 09:00 and 17:00 for 12 weeks. *ALPL*, alkaline phosphatase; *BGLAP*, gamma-carboxyglutamate protein; *COL1A2*, collagen type I alpha 2 chain; MB, medullary bone; *RUNX2*, runt related transcription factor 2

At 6 h post-oviposition, laying hens on the LR phosphorus feeding regimen had: (1) increased MB alkaline phosphatase (*ALPL*) mRNA expression (Fig. 6A, *P *< 0.05) when compared to those on the RR and RL regimens; (2) increased MB runt related transcription factor 2 (*RUNX2*) mRNA expression (Fig. 6B, *P *< 0.05) when compared to those on the RR and LL regimens; (3) increased MB gamma-carboxyglutamate protein (*BGLAP*) mRNA expression (Fig. 6C, *P *< 0.05) when compared to those on the RL and LL regimens. At 18 h post-oviposition, laying hens on the LL phosphorus feeding regimen had increased MB *ALPL* mRNA expression (Fig. 6A, *P *< 0.05) when compared to those on RR, RL and LL regimens. Within the LR phosphorus feeding regimen, laying hens had decreased MB *ALPL, RUNX2, BGLAP* and collagen type I alpha 2 chain (*COL1A2*) mRNA expressions at 18 h post-oviposition when compared to 6 h post-oviposition (Fig. 6A−D).

## Discussion

The phosphate metabolism of laying hens exhibits a characteristic circadian rhythm over the course of the 24-h egg-laying cycle [21]. A nadir of serum phosphate concentration was observed at the time of oviposition (which happened during 07:30−08:30 in the current study). After oviposition, serum phosphate concentration was gradually increased and raised to the peak at 01:30−02:30 in the next morning (18-h after oviposition). The presence of serum phosphate oscillations was initially thought to be a simple result of the eggshell formation cycle [31]. However, recent advances in phosphorus nutrition mechanisms indicting that serum phosphate oscillations may play vital roles in regulating the fast MB remodeling process which directly determined the quality of eggshell formation [32]. What need to be studied is whether the MB remodeling and eggshell formation efficiency could be improved by strengthening the phosphate circadian rhythms, especially when the serum phosphate lost its rhythms in cases of metabolic diseases, aging and nutritional disorders [17, 33, 34]. Indeed, abnormal phosphate rhythm has repeatedly been described as a typical symptom in bone and kidney related diseases in humans and animals [19, 32]. Not surprisingly, the management of phosphate rhythm is difficult, and, despite multifaceted approaches have been tested regarding nutritional intervention [35, 36] and metabolic regulation [37], it remains unsuccessful or at least inefficient. The daily egg-laying physiology makes avian systems excellent models for understanding the underlining mechanisms and developing effective strategies for managing body phosphate rhythms.

Daily oscillation of serum phosphate concentrations is the sum of multi-organ interactions among intestine, kidneys, and bone [38]. What is particular in laying hens is the existence of MB (which provides a rapidly accessible reservoir for calcium and phosphate) [21] and oviduct uterus (which secrets all necessary ionic and organic precursors for the eggshell formation process) [39]. The eggshell formation process can be divided into three stages: initial phase (from 5 to 10 h after oviposition), growth phase (from 10 to 22 h after oviposition) and terminal phase (from 22 to 24 h after oviposition) [40]. In the current study, increased protein productions of calcium tunnels/transporters (i.e., TRPV6 and CaBP-D28k) were observed in oviduct uterus during the eggshell formation period, indicating an increasing of calcium secretion. As a result, serum calcium decreased and the absorption of MB was stimulated. When the eggshell formation was mostly completed in the early morning, serum calcium started to increase as a result of decreased calcium secretion and increased feed intake. Accordingly, the MB was reformed. The above-mentioned changes clearly demonstrated the interactions between MB and body calcium-phosphate nutritional conditions. Of particular note, in this study, serum phosphate oscillation in laying hens was apparently not simply derived by MB remodeling, because kidney protein productions of NPt2a (a major phosphate transporter in the kidney) was significantly increased before serum phosphate concentration reached the peak. Seemly, during the fast-deposition period of eggshell formation, the high-phosphate status in serum was achieved by both MB releasing and kidney resorption. These observations brought up a concept that the increased serum phosphate concentration is physiologically required by the laying hens for eggshell formation. In this sense, improving phosphate circadian rhythms may benefit MB remodeling and eggshell formation.

Manipulating the sequence of nutrient ingestion has been shown as effective in preventing circadian misalignment and metabolic disorders [41]. In the current study, layer on the LR phosphate feeding regimen (fed 0.14% NPP at 09:00 and 0.32% NPP at 17:00) had increased levels of egg specific gravity, shell index, eggshell thickness and eggshell strength, when compared to layers on the RR phosphate feeding regimen (fed 0.32% NPP at both 09:00 and 17:00). Possibly, the LR phosphate feeding regimen induced an increase in uterus TRPV6 protein production, and thereby increased uterus calcium secretion, which directly determines the quality of the fast-deposition eggshell [42]. Impaired eggshell quality was also observed in layers on the LL phosphate feeding regimen, which represent a direct deprivation of dietary inorganic phosphate without considering the body phosphate rhythms. These results support the hypothesis that eggshell quality could be improved by a sequential phosphate feeding regimen that designed to enhancing body phosphate circadian rhythms in aged laying hens. We previously showed that when laying hens were fed with the LR regimen, egg production performance was well supported with significant decreases in phosphorus excretion [43]. Thus, dynamic phosphorus feeding regimen has the potential to be used in commercial laying hen farms. To give concrete conclusions, many more different feeding regimes, with more dietary phosphorus levels and feeding time points, will need to be tested in future studies. In the current study, phosphorus feeding regimens were simply adjusted by the changes in dietary phosphorus levels. Indeed, part of the phosphorus may be given via drinking water [44]. So, it is worth exploring to adjust phosphorus feeding regimens by controlling water phosphorus concentrations. In humans, the sequential nutrition of phosphate has rarely been studied [12, 13]. Even in patients with serious phosphate metabolic diseases, phosphate is simply intervened by controlling overall dietary concentrations [45, 46]. Future studies will need to further illustrate the mechanisms and potential applications of sequential phosphate regimens in humans and animals.

MB is a special bone tissue that forms in the bone marrow cavities of egg-laying birds which provides calcium for eggshell formation [40]. In the present study, layers on the LR phosphate feeding regimen had increased MB remodeling ability (shown as increased absorption rate during eggshell formation period), when compared to those on all the other phosphate regimens. These results well explained the increased shell quality in layers on the LR phosphate feeding regimen. In retrospect, the importance of phosphate nutrition in regulating bone remodeling has been well documented in humans and animals [47, 48]. The fact that daily dynamic phosphate feeding regimen regulated MB remodeling further suggesting the possibility of using sequential phosphate nutrition technologies in control bone mineralization diseases [49]. In humans and animals, bone remodeling markers exhibit typical diurnal variations, and circadian disruption (by sleep alterations or metabolism disorders) is associated with the bone remodeling disorder osteoporosis [50, 51]. Especially, considering the circadian disruption and progressive bone loss in aged individuals, strategies to strengthen intrinsic calcium-phosphate circadian rhythms are highly warranted [49, 52]. Such strategies should preferably involve nutritional interventions (e.g., daily dynamic dietary regimens) instead of pharmaceutical drugs that are costly and may cause adverse effects [41, 53].

## Conclusion

In conclusion, we demonstrate that a simple daily dynamic phosphate feeding regimen (i.e., fed 0.14% NPP at 09:00 and 0.32% NPP at 17:00), which was designed to strengthen intrinsic phosphate circadian rhythms, enhanced MB remodeling, elevated oviduct uterus calcium secretion, and subsequently increased eggshell quality in laying hens. These results underscore the importance of manipulating the sequence of phosphate ingestion, instead of simply controlling dietary phosphate, in modifying the bone remodeling process.

## Supplementary Information

**Additional file 1:** **Table S1.** Sequences of primers used for the quantitative real-time PCR analysis^1^.

## Data Availability

The data produced or analyzed during the current study are available from the corresponding author by reasonable request.

## Abbreviations

ACTB: β-actin
ALPL: Alkaline phosphatase
BGLAP: Gamma-carboxyglutamate protein
CaBP-D28k: Calbindin D‐28k
COL1A2: Collagen type I alpha 2 chain
CTX-I: C-terminal telopeptide of type I collagen
MB: Medullary bone
NPP: Non-phytate phosphorus
NPt2a: Type 2a sodium-phosphate co-transporters
PiT1: Type III sodium-dependent phosphate transporter 1
PiT2: Type III sodium-dependent phosphate transporter 2
RUNX2: Runt related transcription factor 2
TRAP: Tartrate-resistant acid phosphatase
TRPV6: Transient receptor potential vanilloid 6
