# Supplementary material for: Adjusting phosphate feeding regimen according to daily rhythm increases eggshell quality via enhancing medullary bone remodeling in laying hens
Source: J Anim Sci Biotechnol. 2023 Mar 10;14:17. doi: 10.1186/s40104-023-00829-0 (PMC9999492; doi:10.1186/s40104-023-00829-0)
Supplement: Supplementary file 1 — Additional file 1: Table S1. Sequences of primers used for the quantitative real-time PCR analysis1. [file 40104_2023_829_MOESM1_ESM.docx]

**Table S1** Sequences of primers used for the quantitative real-time PCR analysis^1^

| Gene | Accession no. | Primer sequences (5'→3')^2^ | Product length, bp |
| --- | --- | --- | --- |
| *ALPL* | NM_205360.1 | F: AACGTCAGCCGTGTGGACTTC | 78 |
|  |  | R: CTCGTTGTTCCTGTCCAGCTCATA |  |
| *RUNX2* | [NM_204128.1](https://www.ncbi.nlm.nih.gov/entrez/viewer.fcgi?db=nucleotide&id=45383853) | F: AGAACCAGGTGGCCAGATTCA | 134 |
|  |  | R: CGTCACCTTTATGGCTCTGTGGTA |  |
| *BGLAP* | [NM_205387.3](https://www.ncbi.nlm.nih.gov/entrez/viewer.fcgi?db=nucleotide&id=1417767755) | F: TCGCAGTGCTAAAGCCTTCAT | 137 |
|  |  | R: GCTCACACACCTCTCGTTGG |  |
| *COL1A2* | [NM_001079714.2](https://www.ncbi.nlm.nih.gov/entrez/viewer.fcgi?db=nucleotide&id=206597433) | F: GGCTTTGATGCAGAATACTACCG | 90 |
|  |  | R: GTTGTTCAATGTTTTCAGAGTGGC |  |
| *ACTB* | [XM_029084520.1](https://www.ncbi.nlm.nih.gov/entrez/viewer.fcgi?db=nucleotide&id=1631901320) | F: ATTGTCCACGCAAATGCTTC | 173 |
|  |  | R:AAATAAAGCCATGCCAACTCGTC |  |

^1^*ACTB*, β-actin; *ALPL*, alkaline phosphatase; *BGLAP*, gamma-carboxyglutamate protein; *COL1A2*, collagen type I alpha 2 chain; *RUNX2*, runt related transcription factor 2

^2^ F, forward; R, reverse
